# Estimating Heritabilities and Genetic Correlations: Comparing the ‘Animal Model’ with Parent-Offspring Regression Using Data from a Natural Population

**DOI:** 10.1371/journal.pone.0001739

**Published:** 2008-03-05

**Authors:** Mikael Åkesson, Staffan Bensch, Dennis Hasselquist, Maja Tarka, Bengt Hansson

**Affiliations:** Department of Animal Ecology, Lund University, Lund, Sweden; University of Edinburgh, United Kingdom

## Abstract

Quantitative genetic parameters are nowadays more frequently estimated with restricted maximum likelihood using the ‘animal model’ than with traditional methods such as parent-offspring regressions. These methods have however rarely been evaluated using equivalent data sets. We compare heritabilities and genetic correlations from animal model and parent-offspring analyses, respectively, using data on eight morphological traits in the great reed warbler (*Acrocephalus arundinaceus*). Animal models were run using either mean trait values or individual repeated measurements to be able to separate between effects of including more extended pedigree information and effects of replicated sampling from the same individuals. We show that the inclusion of more pedigree information by the use of mean traits animal models had limited effect on the standard error and magnitude of heritabilities. In contrast, the use of repeated measures animal model generally had a positive effect on the sampling accuracy and resulted in lower heritabilities; the latter due to lower additive variance and higher phenotypic variance. For most trait combinations, both animal model methods gave genetic correlations that were lower than the parent-offspring estimates, whereas the standard errors were lower only for the mean traits animal model. We conclude that differences in heritabilities between the animal model and parent-offspring regressions were mostly due to the inclusion of individual replicates to the animal model rather than the inclusion of more extended pedigree information. Genetic correlations were, on the other hand, primarily affected by the inclusion of more pedigree information. This study is to our knowledge the most comprehensive empirical evaluation of the performance of the animal model in relation to parent-offspring regressions in a wild population. Our conclusions should be valuable for reconciliation of data obtained in earlier studies as well as for future meta-analyses utilizing estimates from both traditional methods and the animal model.

## Introduction

A main aim in evolutionary biology is to predict phenotypic change enforced by natural and sexual selection. This requires, among other things, detailed knowledge about the inheritance of phenotypic traits. Traditionally, heritabilities have been estimated by correlations of close kin, e.g. parent-offspring regressions [1]–[4]. During the last decade, the study of evolutionary quantitative genetics in wild populations has made a transition from the traditional use of close-kin comparisons to the more powerful ‘animal model’ using restricted maximum likelihood (REML) [4], [5] to estimate quantitative genetic parameters in natural populations [6]. An animal model takes into account all relationships in a pedigree and is therefore expected to provide estimates of quantitative genetic parameters with higher precision than estimates restricted to the similarity between close kin. It is also less likely to be biased by complicating factors such as assortative mating, inbreeding, selection and shared environment [7]. Moreover, the animal model is expected to be statistically more robust to unbalanced data sets compared to parent-offspring models.

A recent review [6] found and compared published data on heritabilities, estimated from the same populations by both the animal model and parent-offspring regressions. The comparison included heritabilities of 11 traits from 6 species. The pattern emerging from these studies is that heritabilities and standard errors are generally lower with animal models than with parent-offspring regression [6]. However, when comparing results of the two methods based on published data one is confronted with several problems. First, the estimates are mostly taken from data that differ in sample size; e.g. larger data sets are frequently accessible in later publications using the animal model technique. Second, comparisons could be hampered, because information about variances and means of traits are lacking in many, in particular older studies [8]. Third, many animal model analyses use individual repeated measures [9], [10] instead of mean trait values that is used in parent-offspring regressions. The within-individual variation is partly due to phenotypic plasticity and partly due to measurement error. The rational behind using mean traits in parent-offspring regressions has been to avoid pseudo-replication and to account for measurement errors. Thus, when comparing (repeated measures) animal model estimates with estimates derived from mean trait values, one needs to take into account that within-individual variance is likely affecting the estimate of phenotypic variance and possibly also the additive and residual variance [11].

Given that heritability estimates for the vast majority of species and traits still come from parent-offspring analyses [3] it would be valuable to evaluate the accuracy of this method compared to the animal model technique. There are to our knowledge only two published studies directly comparing and evaluating the animal model and parent-offspring regression techniques using the same data sets from natural populations [12], [13]. The results in one of these studies, on long-tailed tits (*Aegithalos caudatus*) [13], are in line with the general conclusions in Kruuk's review [6] which include lower heritability accompanied with smaller standard error when using the animal model. In a study of bighorn sheep (*Ovus canadensis*), the maternal-offspring heritability of age class-specific body mass was similar to the corresponding animal model heritability for older ages, but lower for early age-classes along with only moderate reductions in standard errors [12]. Even though these results were not in line with Kruuk's conclusions, the comparison between the two methods in the sheep study was limited by the fact that maternal effects were not fully accounted for in the animal model since all father identities were unknown. Nevertheless, evaluations like these are important in order to reconcile results in studies using different methods. Moreover, there is a need for understanding why these methods may produce different results; whether it could be an effect of including more extended pedigree information or due to replicated sampling from the same individuals in the animal model analyses.

In the present study, we compare different methods to estimate heritabilities and genetic correlations using data from eight morphological traits collected in a natural population of great reed warblers (*Acrocephalus arundinaceus*). We aimed at evaluating whether the estimated parameters differed when employing the different methods to the same data set, and if so to understand the underlying causes. Our study population has been monitored for more than twenty years [14]–[16] and we have access to a large pedigree in which parentage has been resolved with molecular techniques for the majority of individuals [17]–[19]. We therefore expect animal models to be more powerful when estimating quantitative genetic parameters than parent-offspring regressions. Furthermore, there are characteristics of the population that may violate the assumptions of parent-offspring regressions, i.e. some traits are subjected to directional or stabilizing selection [20], some show influence of shared environment between parents and offspring [20], and there are records of relatively high linkage disequilibrium throughout the genome in great reed warblers ([21], Hansson, B and Csilléry, K unpublished).

To separate between the effects of utilizing all relationships in a pedigree when estimating heritability from the effects of using multiple measurements of the same individual, we used two animal models for each trait and trait combination. In the first animal model, we used the arithmetic mean of all measures of an individual and compared this with the parent-offspring model to investigate how bias and precision are affected by the use of all relationships in the pedigree. In the second model, we used repeated measures of the same individual, thereby producing estimates that may not only be influenced by the use of a larger pedigree but also by information about variation within individuals.

## Methods

### Study species and morphometrics

The great reed warbler (*Acrocephalus arundinaceus*) is a large-sized warbler belonging to the family Sylviidae [22]. It winters in sub-Saharan Africa and migrates to breed in reed lakes in Eurasia [23]. The great reed warbler has a facultative socially polygynous breeding system and about 40 % of the territorial males form social pair bonds with 2–5 females in a season [24]. The breeding population at Lake Kvismaren (50°10′N, 15°25′E) has been monitored since 1983 [14]–[16], [24]. Almost all breeding adults and un-paired males have been captured in mist-nets and then colour-ringed, measured for morphological traits, weighed and blood sampled. Located nests were visited every third day until chicks fledged (when 14–16 days old). When about nine days old, chicks were ringed, measured and blood sampled.

We have taken blood samples from almost all adults and nestlings in the study area since 1987. True parentage of more than eighty percent of these individuals has been assigned with minisatellite DNA fingerprinting [17] or microsatellite genotyping ([18], [19], unpublished material). The frequency of extra-pair young is ca 3 % in the population and in the following analyses we use the genetic father of all offspring. We estimate that among the non-genotyped families no more than two offspring should be sired by an extra-pair male.

We used data collected between 1983 and 2002. After the founding event in 1978 the population has increased to a size of about 50 adults (range 42–78 since 1989). The major increase in population size occurred between 1983 and 1989 [25].

The pedigree we have used in this study contains 523 adults of which 199 individuals were hatched in Kvismaren and have parents that previously have been caught, ringed and measured. For three individuals, we only know the identities of the fathers whereas the mothers were unringed and thus immigrants providing no morphometric or genetic data. Among the adult great reed warblers in Kvismaren there are 89 sib pairs, 322 half sibs, 94 cousins, 404 parent-offspring pairs, 337 grandparent-grandchild pairs and 145 avuncular pairs (retrieved by PEDSATS 0.6.5 [26]), indicating a rather complex pedigree.

We estimated heritabilities and variance components for wing length [27], wing projection (the distance between the first secondary and the longest primary feather of a relaxed wing), tail length, bill width, bill height, bill length, skull length and tarsus length [20]. Adults were measured for all traits from 1991 and onwards. Before this time we only measured the wing length and tarsus length.

### Parent-offspring regression

We used the same methods as reported in Åkesson *et al.* [20] to estimate (narrow sense) heritabilities and additive variances. Prior to the heritability analyses, we tested for fixed effects on the traits by using a mixed linear model (GLMM) with repeated measurements as a random effect (SAS Proc Mixed; see [28]). Each trait was corrected for age, sex, year and/or ringer identity (Table S1) by subtracting the observed value with the proper fixed effects. We lacked the identity of the ringer for 10 measuring events. To avoid reduction in sample size, we fitted these particular measurements with a dummy ringer before the mixed linear model analyses. We calculated heritabilities of the eight traits by regressing the average offspring trait values on average parent values, henceforth referred to as the parent-offspring model. We used the average value of full-sibs to avoid pseudo-replication in the regression analysis. The estimated heritability corresponds to the slope of the midparent-midoffspring regression [11].

### Animal model analyses

Heritabilities and variance components of the phenotypic variance were estimated with restricted maximum likelihood (REML) models, which are preferred over maximum likelihood models when fitting a large number of fixed effects [4]. The program we used was ASReml 2.0 [29]. We fitted animal models with random effects and fixed effects: **y** = ***X*** **b**+***Z***_a_ **a**+***Z***_c_ **c**+***Z***_m_ **m**+***Z***_n_ **n**+**e**, where **y** is the vector of observed phenotypic values of the individuals and vectors **b** = fixed effects, **a = **additive effects, **c = **permanent environment effects, **m = **maternal effects, **n = **common-nest effects and **e = **residual effects. ***X***, ***Z***_a_, ***Z***_c_, ***Z***_m_ and ***Z***_n_ are design matrices relating the records to the appropriate fixed and random effects [4]. We collectively refer **c**, **m**, **n** and **e** as environmental effects and their variance as environmental variance. Note that the use of this terminology does not exclude the possibility that they all may incorporate different sources of (non-additive) genetic effects. The repeated measurements of the same individual will group into the permanent environment effects and is likely to incorporate environmental effects that has a long-term effect (e.g. maternal, dominance, epistasis and cohort effects) on an individual [6]. Maternal effects will group individuals with the same mother and common-nest effects will group those raised in the same nest. To avoid sample size loss due to missing information about mother and nest identity, we fitted unique dummy values to each individual with a missing value. These individuals are almost exclusively immigrants and are therefore very likely to origin from different mothers and nests. We also conducted analyses after deleting individuals with missing values for random factors (such as those with unknown mothers) and the result was very similar but the parameters had larger sampling errors probably due to lower sample size (data not reported in this study).

We used two different animal model approaches to estimate heritabilities and variance components. In the first animal model, we used the mean of the individual trait values. This will henceforth be referred to as the mean traits animal model. The total phenotypic variance (V_P_) was then partitioned into additive genetic variance (V_A_), maternal effect variance (V_M_), common-nest effect variance (V_B_) and residual variance (V_R_). This data set is identical to the data set used for the parent-offspring regression, with exception of the use of average values from individuals in the same brood in the latter method. For comparative purposes we also standardized the phenotypic, additive and environmental variance components of each trait by calculating coefficient of variation (CV), i.e. the square-root of the variance component divided by the non-standardized phenotypic mean (Table S1) of the trait (cf. [8]).

In the second animal model we used repeated measurements (if available) from the same individual, henceforth called repeated measures animal model, and this included fixed effects (instead of corrected values). Thus, V_P_ was partioned into V_A_, variance due to permanent environment effects (V_PE_), V_M_, V_B_ and V_R_ in such a way that V_P_ = V_A_+V_PE_+V_M_+V_B_+V_R_. The narrow-sense heritability was calculated as the ratio of additive variance to the total phenotypic variance: *h* ^2^ = V_A_/V_P_, the permanent environment effect as *c* ^2^ = V_PE_/V_P_, the maternal effect as *m* ^2^ = V_M_/V_P_ and the common-nest effect as *b* ^2^ = V_B_/V_P_. All data from the repeated measures animal model are reported in Table S1.

Three of eight traits had a significant permanent environment effect in the repeated measures animal model (Table S1), ranging between *c* ^2^ = 0.13±0.09 (SE) for tarsus length and 0.45±0.11 (SE) for bill depth. In three cases, the estimates of V_PE_ were locked at the minimum boundary level of the model and no standard errors were returned. In those cases, the estimates were very small or not accompanied with sampling error and we chose not to present the parameters in Table S1. None of the traits had any significant variance due to maternal or common-nest effect. However, we chose to keep maternal effect in tarsus length and common-nest effect in wing length and wing projection in the models for further analyses, to avoid overestimation of the additive effects.

### Repeatabilities

The repeatability (*r* ^2^) of a trait describes the proportion of variance in the trait that is due to variation among rather than within individuals [30]. We calculated repeatabilities from the components of variance extracted from the repeated measures animal model as the sum of the heritability and the portion of phenotypic variance due to any other random effect (e.g. permanent environment effect) if included into the mixed model (Table S1). The repeatabilities ranged between 0.36 and 0.95 with a mean of 0.61 (Table S1). These *r* ^2^s were highly correlated (Pearson correlation: *r* = 0.987, N = 8, *P*<0.001), and showed no significant deviation in sign and magnitude from the *r* ^2^s reported in Åkesson *et al.* [20] that were calculated (in accordance with [30]) by using repeated values corrected for fixed effects (Wilcoxon signed-rank test: *Z*_W_ = 0.84, N = 8, *P* = 0.4). The standard errors of the repeated measures animal model were very similar to the standard errors estimated according to Lessells and Boag's method [31], as indicated by the high correlation (*r* = 0.994, N = 8, *P*<0.001) and non-significant difference in sign and magnitude (*Z*_W_ = 0.84, N = 8, *P* = 0.4) [20]. We therefore chose to report only the animal model estimate of repeatability for each trait (Table S1).

### Genetic and phenotypic correlations

Genetic correlations (*r*_A_) were estimated by regressing average offspring values of trait X on average parent values of trait Y, and vice versa, in accordance with the methods described in [4]. Prior to these analyses, all traits were corrected for significant effects of age, sex, year and ringer (see above). The calculation of *r*_A_ involves dividing the covariances between different traits X and Y (covXY) in parents and offspring with the square-root product of the covariances between the same traits (covXX and covYY, respectively). Since there are two possible products of covXY there are also two estimates of *r*_A_ (*r*_A1_ and *r*_A2_). We present the arithmetic mean of *r*_A1_ and *r*_A2_ [4]. The data used for estimating *r*_A1_ and *r*_A2_ were balanced in the sense that there were no missing values for trait X and Y in neither parents nor offspring. Thus, the calculation of *r*_A1_ and *r*_A2_ for trait X and Y are based on the same individual samples. To estimate the standard error of *r*_A_, we applied the procedures described in Robertson [32] and Falconer and Mackay [11].

Genetic correlations were also estimated with bivariate animal models based on both arithmetic means and repeated measures. The models included the significant fixed effects and random effects for each trait (estimated from univariate models). Genetic correlations were calculated only for traits that were observed to have significant additive genetic variance because *r*_A_ is theoretically undefined when one trait has heritability equal to zero ([4]; see also [10]). We include bill width in the genetic correlations due to its relatively high heritability that tended to be significant. Sample sizes are reported in Table S2.

Phenotypic correlations (*r*_P_) were estimated for each pair of trait as the Pearson product moment correlation coefficient using the mean of the corrected phenotypic values for each individual (see above). Correlation coefficients and their standard errors were extracted from SPSS [33]. Sample sizes are reported in Table S2. We also estimated phenotypic correlations in ASReml for both types of animal models by dividing the phenotypic covariances between the traits with the multiplied standard deviations.

### Statistics

Parent-offspring analyses were conducted in SPSS version 14.0 [33], and the animal models in ASReml 2.0 [29]. In the animal models, the statistical significance of random factors was assessed by comparing the full model with the model without a random factor using the Akaike Information Criteria (see [29] for details). We kept random factors (i.e. maternal and common-nest effects) that affected the component of additive variance even if non-significant to avoid overestimation of the additive variance. Also, non-significant permanent environment effects were kept in the model to avoid the effects of pseudo-replication. The significance of differences between estimates of *h* ^2^, *r* ^2^, *m* ^2^, *b* ^2^ and *r*_A_ from other estimates or from zero was assessed by calculating *z* scores
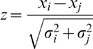
where *x_i_* and *x_j_* are the two different estimates and *σ_i_* and *σ_j_* the respective standard errors. In the case of traits being tested against a value of zero the formula is reduced to the ratio between the estimate and the square-root of its standard error. The corresponding two-tailed significance level for *z* scores were taken from a large sample standard normal distribution.

We compared the two methods to estimate of V_P_, V_A_, V_R_, V_PE_, *h* ^2^ and standard error of *h* ^2^ (SE(*h* ^2^)) using Spearman-rank correlation (*ρ*) and Wilcoxon signed-rank tests (*Z*_W_). We used Wilcoxon signed-rank test and Pearson correlation (*r*) to test for the difference in elements of *r*_A _matrices estimated by the three models. The significance of the Pearson correlation coefficient between *r*_A_ matrices was tested by using a resampling procedure (Mantel test; [34]). The values of the two matrices were randomized N = 10,000 times and correlation coefficients calculated for each randomization were collected. The significance level is given by (n+1)/(N+1), were n is the number of randomized values that are equal to or more extreme than the observed correlation.

## Results

### Comparing parent-offspring regression and mean traits animal model

There were significant heritabilities for 7 of the 8 traits, ranging between 0.39 and 0.97 for the parent-offspring model and between 0.32 and 0.84 for the animal model (Table 1). Bill depth heritability was non-significant for both methods (parent-offspring model; *h* ^2^ = 0.07±0.16 SE; mean traits animal model: *h* ^2^ = 0.06±0.12 SE).

**Table 1:** Heritabilities (*h* ^2^) and corresponding standard errors (SE) of eight morphological traits estimated from the different models.

Trait	h*^2^ (SE)*		
	Parent-offspring model	Mean traits animal model	Repeated measures animal model
Wing length	0.762 (0.092)^***^	0.716 (0.107)^***^	0.674 (0.082)^***^
Wing projection	0.468 (0.140)^***^	0.477 (0.330)	0.267 (0.093)^**^
Tail length	0.677 (0.115)^***^	0.808 (0.086)^***^	0.551 (0.050)^***^
Bill depth	0.066 (0.164)	0.065 (0.122)	0.054 (0.098)
Bill width	0.390 (0.123)^**^	0.461 (0.137)^***^	0.200 (0.114)^†^
Bill length	0.974 (0.114)^***^	0.836 (0.077)^***^	0.717 (0.034)^***^
Skull length	0.435 (0.137)^**^	0.322 (0.130)^*^	0.326 (0.113)^**^
Tarsus length	0.724 (0.107)^***^	0.727 (0.112)^***^	0.711 (0.084)^***^

Two-tailed significances of the heritabilities are indicated as ^***^, ^**^, ^*^, ^†^ corresponding to *P*<0.001, *P*<0.01, *P*<0.05 and *P*<0.10 respectively.

The heritabilities of the two methods were highly correlated (Spearman rank correlation: *ρ* = 0.88, N = 8, *P* = 0.004) and did not differ significantly in magnitude (Wilcoxon sign rank test: *Z*_W_ = 0.14, N = 8, *P* = 0.89; Table 2). The differences in *h* ^2^ (parent-offspring *h* ^2^ minus mean traits animal model *h* ^2^) ranged between −0.13 and 0.14. When analysing each trait separately there was no significant differences in *h* ^2^ (range *z* = 0.003–1.00; range *P* = 0.32–0.997). Standard errors of heritabilities (SE(*h* ^2^)) from the two methods tended to be significantly correlated (*ρ* = 0.64, N = 8, *P* = 0.09) and the mean traits animal model generated on average 10.9 % higher standard error than those of parent-offspring (mean traits animal model: mean SE(*h* ^2^) 0.14±0.08 SD; parent-offspring model: mean SE(*h* ^2^) 0.12±0.02 SD), but over all traits the standard error of the two models did not differ significantly (*Z*_W_ = 0.28, N = 8, *P* = 0.78; Table 2).

**Table 2:** Correlations and overall differences between parent-offspring estimates of variance components and heritabilities and corresponding estimates generated from two animal models differing in use of replicated values or individual means.

Parameters	Parent-offspring model vs.			
	Mean traits animal model		Repeated measures animal model	
	Correlation1)	% difference (Z_w_)2)	Correlation1)	% difference (Z_w_)2)
V_P_	1.00^***^	−1.1 (2.10^*^)	1.00^***^	−6.0 (2.52^*^)
V_A_	1.00^***^	−0.7 (0.14^ns^)	1.00^***^	9.6 (2.10^*^)
V_PE_+V_M_+V_B_+V_R_	0.83^*^	−5.9 (1.12^ns^)	0.88^**^	−29.5 (2.52^*^)
*h* ^2^	0.881^**^	1.9 (0.14^ns^)	0.95^***^	22.1 (2.52^*^)
SE of *h* ^2^	0.64^†^	−10.9 (0.28^ns^)	0.59^ns^	32.6 (2.52^*^)

1) Spearman-rank correlation tests were used to test the correlation (*ρ*) between the estimates. Two-tailed significances of are indicated as ^***^, ^**^, ^*^, ^†^ corresponding to *P*<0.001, *P*<0.01, *P*<0.05 and *P*<0.10 respectively.

2) The difference in magnitude between parent-offspring and animal model estimates were tested with Wilcoxon signed-rank tests. Alongside the Wilcoxon signed-rank Z statistic (Z_W_) and significance, we report the difference between the average animal model and average parent-offspring estimates in relation to the average parent-offspring estimate (in percent). For comparative purposes the variance components have been transformed to coefficients of variation (see methods) prior to the calculation of the percentages.

**Figure 1:**
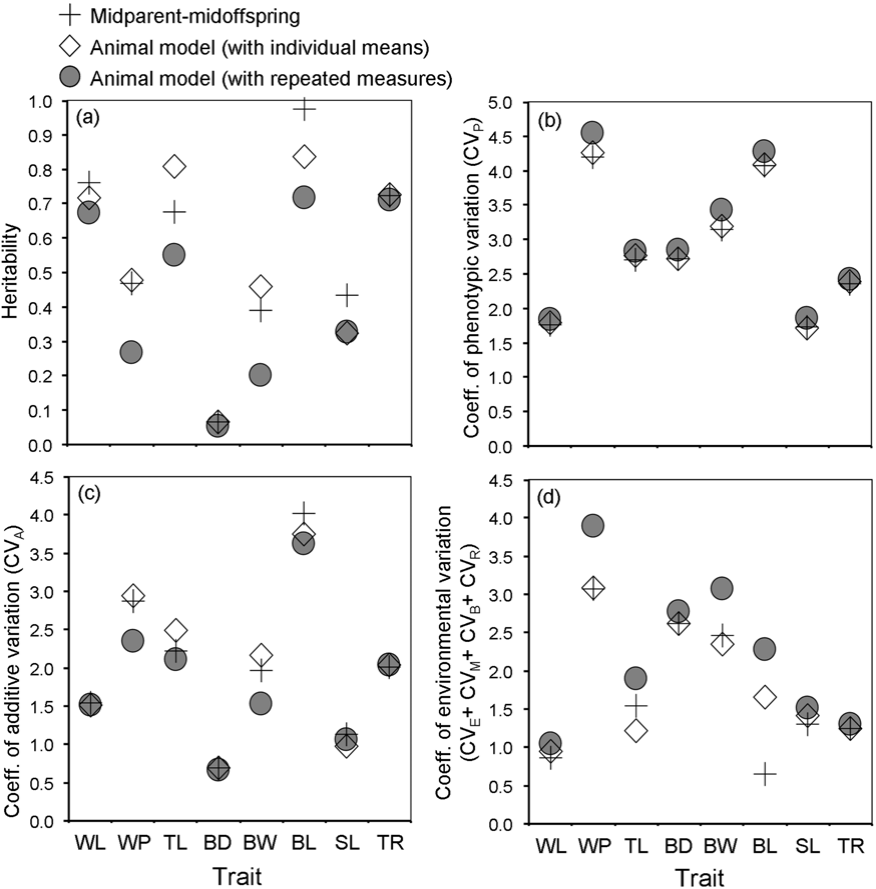
Estimates of heritability (a) and coefficients of variation from three variance components, phenotypic variance (b), additive variance (c) and environmental variance (d), for eight morphological traits. Each component was estimated from parent-offspring regression, mean traits animal model and repeated measures animal. WL = wing length; WP = wing projection; TL = tail length; BD = bill depth; BW = bill width; BL = bill length; SL = skull length; TR = tarsus length.

The phenotypic variance (V_P_) calculated using mean trait animal model was larger in seven of eight traits but very similar in magnitude (Table 2) compared to V_P_ calculated using parent-offspring regression (*Z*_W_ = 2.10, N = 8, *P = *0.034; Figure 1).

The two methods produced additive variances (V_A_) that were highly correlated (*ρ* = 1.00, N = 8, *P*<0.001) and did not differ significantly in magnitude (*Z*_W_ = 0.14, N = 8, *P*  = 0.89; Table 2). The sum of the environmental variance components (V_PE_+V_M_+V_B_+V_R_) for each trait was significantly correlated between the two techniques (*ρ* = 0.83, N = 8, *P* = 0.010) and the difference in magnitude was non-significant (*Z*_W_ *= *1.12, N = 8, *P = *0.26).

There was no significant maternal effect in any of the investigated traits. For all traits except tarsus length the maternal effect was locked at a minimum value (Table S1). Wing projection had a significant variance component due to a common-nest effect (V_B_ = 0.66±0.16). However, due to a large standard error, the ratio between V_B_ and the phenotypic variance (*b* ^2^ = 0.40±0.31) was non-significant (*z* = 1.28, *P* = 0.2). Also, wing length showed a common-nest effect variance (V_B_ = 0.22±0.23), however it did not differ significantly from zero (Table S1). The additive variance was affected very mildly by the incorporation of maternal and common-nest effects and the major part of these environmental components was extracted from the residual variance (data not reported).

### Comparing parent-offspring regression and repeated measures animal model

Six of eight morphological traits that were estimated by repeated measures animal model showed significant additive variance (Table S1). The significant *h* ^2^s ranged from 0.27 to 0.72 with a mean of 0.54. The *h* ^2^ of 0.20 of bill width tended towards significance (*z* = 2.33, *P = *0.08) whereas the *h* ^2 ^of 0.05 in bill depth was far from significant (*z* = 0.60, *P* = 0.58).

The *h* ^2^s from the repeated measures animal model were numerically lower than the *h* ^2^s calculated from parent-offspring regression in all the 8 traits (Table 1), but only significantly so for bill length (*z* = 2.16, *P* = 0.031). The difference between *h* ^2^ from the parent-offspring model and *h* ^2^ from the repeated measures animal model ranged between 0.01 and 0.26, corresponding to an average difference of 22.1 % of the parent-offspring estimate (repeated measures animal model: *h* ^2^ = 0.44±0.24 SD; parent-offspring model: *h* ^2^ = 0.56±0.24 SD; *Z*_W_ *= *2.52, N = 8, *P* = 0.012; Table 2). The SE(*h* ^2^) estimated from the repeated measures animal model was lower for all traits compared to those of the parent-offspring model and differed on average 32.6 % (repeated measures animal model: mean SE(*h* ^2^) 0.08±0.03 SD; parent-offspring model: mean SE(*h* ^2^) 0.12±0.02 SD; *Z*_W_ = 2.52, N = 8, *P* = 0.012; Table 2).

The phenotypic variance (V_P_) calculated using repeated measures animal model was larger in all 8 traits compared to V_P_ calculated using parent-offspring regression (*Z*_W_ = 2.52, N = 8, *P = *0.012; Table 2). The higher V_P_ of the repeated measures animal model was caused by an increase in environmental variance (*Z*_W_ = 2.52, N = 8, *P* = 0.012; Figure 1). For all traits, except tail length, the major part of the increased environmental variance was due to the permanent environment variance, maternal effect variance and common-nest effect variance (Table S1). The higher phenotypic variance obtained when using repeated measures animal model was also a consequence of increased residual variance (V_R_) in wing projection, tail length and bill length.

The repeated measures animal model generated V_A _values that were highly correlated with V_A _values from the parent-offspring model (*ρ* = 1.00, N = 8, *P*<0.001; Table 2). For 7 of 8 traits, the V_A_ was lower in the repeated measures animal model compared to parent-offspring model resulting in a significant overall difference in magnitude (repeated measures animal model: V_A_ = 0.75; parent-offspring model: V_A_ = 0.83; *Z*_W = _2.10, N = 8, *P* = 0.036).

### Comparing trait correlations

**Figure 2:**
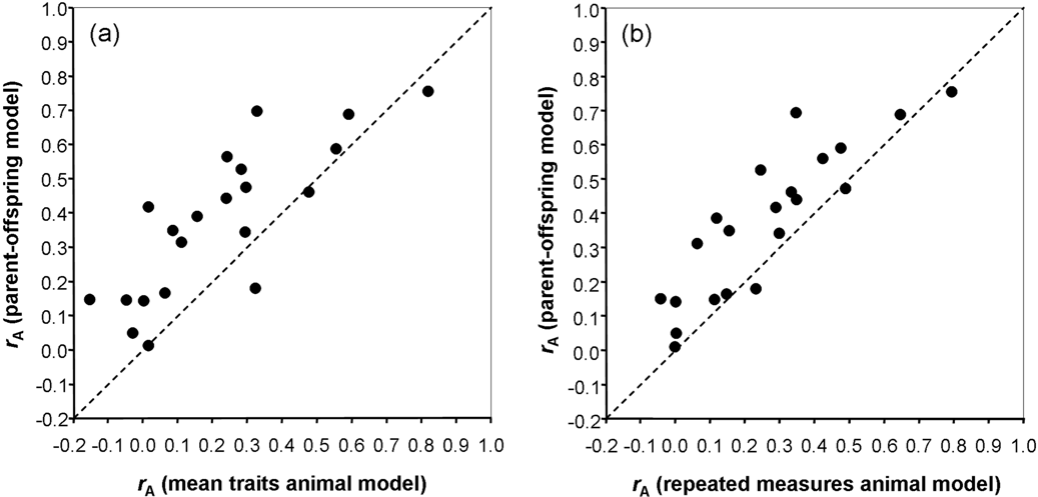
Association between genetic correlations estimated from mean traits animal model and parent-offspring regression (a); and repeated measures animal model and mean traits animal model (b). The dashed line represents the 1∶1 relationship.

Genetic correlations (*r*_A_) calculated with the parent-offspring model were significant in 9 of 21 cases and positive in all cases and the significant *r*_A _values ranged between 0.34 and 0.75 (Table 3). The mean traits animal model gave *r*_A _values that were positive in 18 of 21 cases and the six significant estimates ranged between 0.29 and 0.81 (Table 3). The two methods provided values of *r*_A_ that were significantly correlated between corresponding trait-pairs (Pearson correlation: *r* = 0.81, N = 21; Mantel test: *P*<0.001; Figure 2). For 18 of 21 trait combinations higher estimates of *r*_A_ were calculated with the parent-offspring method (mean traits animal model: mean *r*_A_ = 0.22±0.24 SD; parent-offspring model: mean *r*_A_ = 0.37±0.22 SD; *Z*_W_ *= *3.39, N = 21, *P*<0.001). The standard errors of *r*_A_ generated from the two methods was highly correlated (*r* = 0.904, N = 21; *P*<0.001) and the mean traits animal model generated overall (19 of 21 cases) smaller standard errors (mean traits animal model: SE(*r*_A_) = 0.16±0.05 SD; parent-offspring model: SE(*r*_A_) = 0.19±0.07 SD; *Z*_W_ = 3.60, N = 21, *P*<0.001).

**Table 3:** Phenotypic correlations (above the diagonal), additive genetic correlations (below the diagonal) among seven morphological traits in the great reed warbler, estimated from (a) parent-offspring regression (b) bivariate animal models using individual mean values and (c) bivariate animal models using repeated measures from the same individual.

	Wing length	Wing projection	Tail length	Bill width	Bill length	Skull length	Tarsus length
(a) Parent-offspring model1)							
Wing length	-	**0.49 (0.05)**	**0.57 (0.04)**	0.06 (0.05)	0.08 (0.05)	**0.19 (0.06)**	**0.12 (0.05)**
Wing projection	**0.69 (0.13)**	-	**0.18 (0.05)**	0.002 (0.05)	0.03 (0.05)	**0.15 (0.06)**	**0.14 (0.05)**
Tail length	**0.75 (0.10)**	**0.46 (0.23)**	-	0.07 (0.05)	0.04 (0.05)	**0.16 (0.06)**	0.05 (0.05)
Bill width	0.31 (0.20)	0.41 (0.32)	0.35 (0.21)	-	**0.34 (0.05)**	0.11 (0.06)	**0.18 (0.05)**
Bill length	0.05 (0.14)	0.15 (0.20)	0.16 (0.14)	**0.47 (0.17)**	-	-0.02 (0.06)	**0.20 (0.05)**
Skull length	0.38 (0.20)	0.18 (0.38)	**0.59 (0.19)**	0.44 (0.30)	0.15 (0.20)	-	**0.37 (0.05)**
Tarsus length	0.14 (0.13)	**0.52 (0.19)**	0.01 (0.16)	**0.56 (0.18)**	**0.34 (0.12)**	**0.69 (0.17)**	-
(b) Mean traits animal model1)							
Wing length	-	**0.49 (0.04)**	**0.58 (0.04)**	0.05 (0.06)	0.05 (0.06)	**0.19 (0.06)**	0.10 (0.05)
Wing projection	**0.59 (0.12)**	-	0.17 (0.05)	-0.01 (0.06)	0.000 (0.06)	**0.14 (0.06)**	**0.14 (0.05)**
Tail length	**0.81 (0.07)**	**0.48 (0.18)**	-	0.06 (0.06)	0.01 (0.06)	0.16 (0.06)	0.06 (0.06)
Bill width	0.11 (0.18)	0.02 (0.23)	0.09 (0.16)	-	**0.34 (0.05)**	0.11 (0.06)	**0.17 (0.05)**
Bill length	−0.03 (0.11)	−0.04 (0.15)	0.06 (0.11)	**0.30 (0.14)**	-	−0.03 (0.06)	**0.21 (0.05)**
Skull length	0.16 (0.21)	0.32 (0.27)	**0.56 (0.19)**	0.24 (0.28)	−0.15 (0.18)	-	**0.35 (0.05)**
Tarsus length	0.003 (0.11)	0.28 (0.16)	0.02 (0.12)	0.24 (0.16)	**0.29 (0.10)**	0.32 (0.19)	-
(c) Repeated measures animal model1)							
Wing length	-	**0.46 (0.04)**	**0.58 (0.04**	0.04 (0.05)	0.05 (0.06)	**0.20 (0.06)**	**0.10 (0.05)**
Wing projection	**0.65 (0.12)**	-	**0.20 (0.06**	0.005 (0.06)	0.03 (0.06)	**0.13 (0.06)**	**0.12 (0.04)**
Tail length	**0.79 (0.07)**	0.33 (0.17)	-	0.06 (0.05)	0.01 (0.05)	**0.14 (0.05)**	0.06 (0.05)
Bill width	0.06 (0.23)	0.29 (0.35)	0.16 (0.22)	-	**0.35 (0.04)**	**0.11 (0.05)**	**0.19 (0.05)**
Bill length	0.004 (0.12)	0.11 (0.16)	0.15 (0.12)	**0.49 (0.19)**	-	−0.07 (0.05)	**0.22 (0.05)**
Skull length	0.12 (0.20)	0.23 (0.26)	**0.48 (0.18)**	0.35 (0.33)	−0.04 (0.19)	-	**0.33 (0.05)**
Tarsus length	0.002 (0.11)	0.25 (0.16)	0.001 (0.13)	0.42 (0.24)	**0.30 (0.11)**	**0.35 (0.17)**	-

1) Standard errors of correlations are given in parentheses and significant parameters are written in bold. The sample sizes are reported in Table S2.

Phenotypic correlations between mean trait values within individuals (*r*_P_^*^) were positive in 20 of 21 cases and the 12 significant *r*_P_*-values ranged between 0.11 and 0.57 (Table 3). These data are very similar to the results obtained when using the mean traits animal model approach. Phenotypic correlation calculated with the mean traits animal model was highly correlated with *r*_P_^*^ for each trait pair (*r*_ = _0.997, N = 21; *P<*0.001), but with a slight downward bias (mean traits animal model: mean *r*_P_ = 0.16±0.16 SD; mean *r*_P_^*^ = 0.17±0.16 SD; *Z*_W_ = 2.14, N = 21, *P* = 0.033).

Genetic correlations calculated from the repeated measures animal model were positive in 20 of 21 cases and significant in 6 of them (Table 3). Estimates of *r*_A_ from the parent-offspring model and *r*_A_ from repeated measures animal model were significantly correlated (*r = *0.87, N = 21; *P*<0.001; Figure 2). However, in general the repeated measures animal model gave lower *r*_A_s (18 of 21 trait combinations; repeated measures animal model: mean *r*_A_ = 0.26±0.22 SD; parent-offspring model: mean *r*_A_ = 0.37±0.22 SD; *Z*_W_ *= *3.39, N = 21, *P*<0.001), but none of these differences were significant. The SE(*r*_A_)s calculated with repeated measures animal model were lower in 14 of 21 cases and were not significantly different from the SE(*r*_A_)s from the parent-offspring analyses (repeated measures animal model: SE(*r*_A_) = 0.18±0.07 SD; parent-offspring: SE(*r*_A_) = 0.19±0.07 SD; *Z*_W_ = 1.20, N = 21, *P* = 0.23).

Phenotypic correlations calculated from the repeated measures animal model were positive in 20 of 21 cases and the 13 significant estimates ranged between 0.10 and 0.58 (Table 3). The *r*_P_ estimates from the repeated measures animal model correlated significantly with the corresponding *r*_P_^*^ (*r* = 0.99, N = 21; *P*<0.001), but tended towards having lower estimates (repeated measures animal model: mean *r*_P_ 0.16±0.16 SD; mean *r*_P_^*^ = 0.17±0.16 SD; *Z*_W_ = 1.79, N = 21, *P* = 0.07).

## Discussion

We have compared parent-offspring regression estimates of heritability and genetic correlation with estimates obtained when using animal model. To our knowledge this is one of the first and most exhaustive study directly comparing these methods using the same data set in a population of free-ranging animals exposed to its natural environment (cf. [12], [13]).

We found no overall difference in heritabilities and associated standard errors between parent-offspring regression and mean traits animal model even though the latter utilises much more extensive pedigree information than the former. In general, the heritability was very similar for the two methods. This similarity in *h* ^2^ was also reflected by strong correlation in the additive variance (V_A_) as well as in the environmental variance (V_M_+V_B_+V_R_). Hence, the estimation of heritability and additive variance with parent-offspring regression were not seriously biased by, e.g. shared environment between parents and offspring. However, tarsus length and wing projection were the only traits that showed a parsimonious model (based on the Akaike Information Criteria) when including an environmental variance component (Table S1). Despite the maternal effect on tarsus length, the heritabilities were very similar for the two methods (0.73 and 0.72 for animal model and parent-offspring regression, respectively). It is worth noting that tarsus length was the only trait that was found to have significantly higher maternal inheritance than paternal inheritance in previous singleparent-midoffspring regression analyses ([20]; see also [35]). The very similar estimates of heritability of tarsus length would thus suggest that parent-offspring regression is not seriously biased by the common environment shared by the mother and her offspring. However, in great reed warblers, tarsus length was only moderately affected by the mother's identity (*m* ^2^ = 0.12, Table S1) and it is possible that traits with larger environmental variance (such as life-history traits) are subject to larger bias. In a review of fifteen cross-fostering experiments there was little evidence that shared environment between parents and offspring would seriously bias heritabilities [36]. Although the common-nest effect in wing projection is considerable (*b* ^2^ = 0.40±0.31), this does not affect the differences in heritabilities of the two methods (wing projection *h* ^2^: 0.48 vs. 0.47). This is expected since the nest effect is the result of the environmental covariance between offspring from same nests and is not expected to influence the covariance between parents and offspring [11]. The alternative explanation to the high similarity in heritabilities in wing projection calculated from the two methods is that the data-set with individual means do not offer enough power for the animal model to resolve biasing effects on the V_A_ estimate. In a recent study [7] it is highlighted that even a fully specified animal model using considerable pedigree information may produce inflated heritabilities due to common nest effects, when these are considerable. Surprisingly, the standard errors of the heritabilities (SE(*h* ^2^)) were largely unaffected by using the mean traits animal model. The exception was SE(*h* ^2^) of wing projection that opposite to the prediction was more than twice the magnitude for the mean traits animal model.

The repeated measures animal model resulted in lower heritabilities for all traits as compared with results from parent-offspring regression, and the difference in *h* ^2^ ranged between 0.01 and 0.26, including a more than 40 % reduction in *h* ^2^ of wing projection and bill width. Furthermore, the repeated measures animal model gave lower sampling errors for all traits, with an average improved accuracy of 33 %. Two factors contributed to the reduction in heritability. First, the phenotypic variance (V_P_) was larger for the repeated measures animal model as compared with individual means. By using repeated measures a new source of variation is introduced into the model, which is the variation between measuring events of the same individual. This within-individual variance can be caused by a natural variation of a character, e.g. individual variation in development rate and phenotypic plasticity in response to different environmental conditions, but also from measurement errors. The repeatability (*r* ^2^) is the proportion of phenotypic variance that is due to variation within individuals [4], [11]. The repeatability can be used as an indication of how much accuracy in the phenotypic trait might be gained by taking multiple measurements. Highly repeatable traits will only gain marginally in accuracy by using multiple measures, whereas traits with low repeatability may be more accurate if many measurements are taken. This is because the ratio between the phenotypic variance (V_P(*n*)_) derived from a data-set with average phenotypes and the phenotypic variance (V_P_) from a data-set with single measurements from the same population of individuals, depends on the repeatability (*r* ^2^) and number of measuring events (*n*) according to
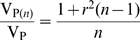

[11]. Thus, for a given *n*, the V_P(*n*)_ is likely to be reduced more in relation to V_P_ if the repeatability is low. As expected, there is a positive correlation (*r* = 0.835, N = 8, *P* = 0.01) between repeatability and the ratio of V_P_ of mean values on repeated measures animal model V_P_ (i.e. V_P(*n*)_/V_P_). Another reason for the lower *h* ^2^ from repeated measures animal models compared to parent-offspring regressions is the former's lower additive variance (V_A_). This may also be a consequence of the within-individual variance as supported by the tendency to a negative correlation between the repeatability (*r*_A_)) and the ratio of the parent-offspring model V_A_ on repeated measures animal model V_A_ (V_A(*n*)_/V_A_) (*r* = −0.70, N = 8, *P* = 0.06). Alternatively, the repeated measures animal model is more powerful in correcting for obscuring effects on the estimation of additive variance. These would be effects that violate the assumptions of the parent-offspring heritabilities such as non-random mating, selection, linkage disequilibrium, epistasis and environmental covariances. We found support for this explanation by the increased accuracy of the heritabilities when using repeated measures animal model compared to the mean traits animal model.

The evolution of a quantitative trait depends on the magnitude of heritability but also on the genetic and environmental correlations with other traits [4], [11], [37]. The genetic correlation shows to what extent two traits have a common genetic background due to pleiotropic effects and linkage disequilibrium (LD; [4]). In the studied great reed warbler population, we have observed a relatively high level of LD ([21], Hansson, B. and Csilléry K, unpublished), which may have to do with the recent founder event and population expansion in the region [25]. The genetic correlations between traits in the population may thus be partly due to LD between genes, partly due to pleiotropy. The genetic correlations estimated from the repeated measures animal model were largely positive, as has previously been observed in natural populations (e.g. [38]–[40], but see [9]).

Large sample sizes are generally required to accurately estimate genetic correlations since they often are subjected to large sampling errors [4], [41]. Also, estimates of genetic correlations from parent-offspring relationships are easily biased by maternal effects and selection [42]. In the present study, we estimated the genetic correlations between 7 traits and compared the parent-offspring approach with the animal model approach (using either individual mean values or repeated measures). These three methods generated highly correlated estimates of the genetic correlations, although there were some differences in overall magnitude. Both animal models generated lower genetic correlations in 18 of 21 trait correlations compared to the parent-offspring model. It is possible that the genetic correlations estimated from the latter model are biased by either shared environment between parents and offspring or by selection acting on the traits (see [20]). That the animal models are less biased by such factors seem at least partly to be explained by the use of a large pedigree, because we observed a similar reduction by both types of animal models. However, standard errors were overall lower only in the animal models using mean values. Apparently repeated measures animal models offer less biased estimates of *r*_A_, but does not manage to reduce the sampling error. Alternatively, the standard errors of genetic correlations from the parent-offspring regression are underestimated. The sampling error of genetic correlations [32], [43] from parent-offspring model is complicated and to a large extent unresolved matter [44]. Simulation studies have shown that the sampling error of genetic correlations may be seriously underestimated for sample sizes under 100, especially if the corresponding heritabilities of the traits are low or genetic correlations are high [45], [46].

### Conclusion

Our results suggest that the increased accuracy of heritability estimates when using animal model is mostly due to the inclusion of repeated measures, and that the heritability estimates appear to be lower when using repeated measures animal models. We did not observe any bias caused by the maternal environment on *h* ^2^, but it should be kept in mind that only one of the 8 investigated traits showed maternal effects. It should also be kept in mind that morphological traits generally show low levels of dominance variance and that our results may not be applicable to other types of traits, such as life-history traits, that are known to be affected to a larger extent by dominance and epistatis [4]. The lower additive variance from the repeated measures animal model is also likely to be due to the within-individual variance of each trait, as indicated by the tendency for a negative correlation between repeatability and ratio of additive variances between the two methods. This implies that additive variances would be overestimated by parent-offspring regression and mean trait animal models when there is natural variation in trait expression within individuals and when there are measurement errors.

Genetic correlations appear to be lower but more accurate (i.e. having lower standard errors) when estimated by the either of the two animal models than by parent-offspring models. This suggests that genetic correlations from parent-offspring models are sensitive to biasing effects such as selection and environmental covariance between relatives, and highlights the importance of taking into account all relatives in a pedigree when estimating genetic correlations.

The reconciliation of results from different studies using different estimation procedures depends on finding out and taking potential methodological discrepancies into account before comparing the data (see e.g. [3], [8]). Only few studies that evaluate the animal model and parent-offspring regressions have been made previously and then on rather limited data [12], [13]. The present study thus provides important knowledge for future meta-analyses aiming at understanding the concept of evolutionary potential.

## Supporting Information

Table S1 The quantitative genetic parameters for eight morphological traits in the great reed warbler estimated from parent-offspring regression, mean traits animal model and repeated measures animal model.
(0.10 MB DOC)

Table S2 Sample sizes for trait correlations and genetic covariances among seven morphological traits in the great reed warbler.
(0.05 MB DOC)
