# Supplementary material for: Estimating Heritabilities and Genetic Correlations: Comparing the ‘Animal Model’ with Parent-Offspring Regression Using Data from a Natural Population
Source: PLoS One. 2008 Mar 5;3(3):e1739. doi: 10.1371/journal.pone.0001739 (PMC2254494; doi:10.1371/journal.pone.0001739)
Supplement: Table S1 — The quantitative genetic parameters for eight morphological traits in the great reed warbler estimated from parent-offspring regression, mean traits animal model and repeated measures animal model. (0.10 MB DOC) [file pone.0001739.s001.doc]

Table S1. The quantitative genetic parameters for eight morphological traits in the great reed warbler estimated from a) parent-offspring regression, b) mean traits animal model and c) repeated measures animal model.

| Trait | Fixed effects^2)^ | Mean | N | V_P_ | V_A_ | V_PE_ | *c*^2^ | V_M_ | *m*^2^ | V_B_ | *b*^2^ | V_R_ | *r*^2^ |
| --- | --- | --- | --- | --- | --- | --- | --- | --- | --- | --- | --- | --- | --- |
| (a) Parent-offspring model^1)^ | | |  |  |  |  |  |  |  |  |  |  |  |
| Wing length | age, sex, year, ringer | 98.24 | 128 | 3.01 | 2.29 | - | - | - | - | - | - | 0.72 | **-** |
| Wing projection | age, sex, year, ringer | 28.86 | 84 | 1.47 | 0.69 | - | - | - | - | - | - | 0.78 | **-** |
| Tail length | age, sex, year | 76.31 | 83 | 4.25 | 2.88 | - | - | - | - | - | - | 1.37 | **-** |
| Bill depth | age, sex, ringer | 5.15 | 84 | 0.02 | 0 | - | - | - | - | - | - | 0.02 | **-** |
| Bill width | year, ringer | 5.07 | 82 | 0.03 | 0.01 | - | - | - | - | - | - | 0.02 | **-** |
| Bill length | sex, year, ringer | 12.54 | 85 | 0.26 | 0.25 | - | - | - | - | - | - | 0.01 | **-** |
| Skull length | sex, year, ringer | 31.11 | 64 | 0.29 | 0.13 | - | - | - | - | - | - | 0.16 | **-** |
| Tarsus length | sex, ringer | 33.15 | 115 | 0.61 | 0.44 | - | - | - | - | - | - | 0.17 | **-** |
| (b) Mean traits animal model^1)^ | | |  |  |  |  |  |  |  |  |  |  |  |
| Wing length | age, sex, year, ringer | 98.24 | 508 | 3.01 (0.19) | 2.22 (0.36) | - | - | 0 | 0 | 0.22 (0.23) | 0.07 (0.08) | 0.66 (0.33) | - |
| Wing projection | age, sex, year, ringer | 28.86 | 375 | 1.47 (0.11) | 0.72 (0.22) | - | - | 0 | 0 | 0.61 (0.16) | 0.40 (0.31) | 0.18 (0.17) | - |
| Tail length | age, sex, year | 76.31 | 376 | 4.25 (0.31) | 3.60 (0.57) | - | - | 0 | 0 | 0 | 0 | 0.86 (0.36) | - |
| Bill depth | age, sex, ringer | 5.15 | 379 | 0.02 (0.00) | 0 | - | - | 0 | 0 | 0 | 0 | 0.02 (0.00) | - |
| Bill width | year, ringer | 5.07 | 376 | 0.03 (0.00) | 0.01 (0.00) | - | - | 0 | 0 | 0 | 0 | 0.01 (0.00) | - |
| Bill length | sex, year, ringer | 12.54 | 379 | 0.26 (0.02) | 0.22 (0.03) | - | - | 0 | 0 | 0 | 0 | 0.04 (0.02) | - |
| Skull length | sex, year, ringer | 31.11 | 308 | 0.29 (0.02) | 0.09 (0.04) | - | - | 0 | 0 | 0 | 0 | 0.19 (0.04) | - |
| Tarsus length | sex, ringer | 33.15 | 456 | 0.61 (0.04) | 0.46 (0.07) | - | - | 0.07 (0.04) | 0.12 (0.09) | 0 | 0 | 0.10 (0.05) | - |
| (c) Repeated measures animal model^1)^ | | | |  |  |  |  |  |  |  |  |  |  |
| Wing length | age, sex, year, ringer | 98.24 | 859 | 3.28 (0.22) | 2.21 (0.36) | 0.27 (0.33) | 0.08 (0.10) | 0 | 0 | 0.24 (0.23) | 0.07 (0.07) | 0.57 (0.04) | 0.82 (0.02) |
| Wing projection | age, sex, year, ringer | 28.86 | 583 | 1.72 (0.11) | 0.46 (0.17) | 0 | 0 | 0 | 0 | 0.16 (0.15) | 0.09 (0.09) | 1.10 (0.10) | 0.36 (0.06) |
| Tail length | age, sex, year | 76.31 | 582 | 4.67 (0.33) | 2.57 (0.37) | 0 | 0 | 0 | 0 | 0 | 0 | 2.10 (0.20) | 0.55 (0.05) |
| Bill depth | age, sex, ringer | 5.15 | 586 | 0.02 (0.00) | 0.001 (0.00) | 0.01 (0.00) | 0.45 (0.11) | 0 | 0 | 0 | 0 | 0.01 (0.00) | 0.50 (0.06) |
| Bill width | year, ringer | 5.07 | 583 | 0.03 (0.00) | 0.01 (0.00) | 0.01 (0.00) | 0.27 (0.12) | 0 | 0 | 0 | 0 | 0.02 (0.00) | 0.47 (0.06) |
| Bill length | sex, year, ringer | 12.54 | 591 | 0.29 (0.02) | 0.21 (0.02) | 0 | 0 | 0 | 0 | 0 | 0 | 0.08 (0.01) | 0.72 (0.03) |
| Skull length | sex, year, ringer | 31.11 | 470 | 0.33 (0.02) | 0.11 (0.04) | 0.06 (0.04) | 0.17 (0.12) | 0 | 0 | 0 | 0 | 0.17 (0.02) | 0.50 (0.06) |
| Tarsus length | sex, ringer | 33.15 | 752 | 0.64 (0.05) | 0.46 (0.07) | 0.09 (0.06) | 0.13 (0.09) | 0.07 (0.05) | 0.10 (0.07) | 0 | 0 | 0.03 (0.00) | 0.95 (0.01) |

1. Estimates of unstandardized population mean values are reported. The variance components reported is the phenotypic variance (V_P_), additive variance (V_A_), residual variance (V_R_), variance due to maternal effects (V_M_) and variance due to the shared environment between offspring in the same nest (V_B_). The repeatability *r*^2^ is also reported for the repeated measures animal model. All estimates are fitted with the corresponding standard error and reported within brackets (if available). The sample sizes (N) correspond to the number of parent-offspring comparisons available and the total number of measurements for the parent-offspring model and animal models, respectively.
2. Each trait are tested for and standardized for the significant effects of age (three levels), sex (two levels), year (twenty levels), ringer (thirty levels), For parent-offspring regressions and mean trait animal models the traits are standardized for the fixed effects before the analyses, while unstandardized traits are analysed in the repeated measures animal models with the corresponding fixed effects included in the models.
