# Supplementary material for: Estimating Heritabilities and Genetic Correlations: Comparing the ‘Animal Model’ with Parent-Offspring Regression Using Data from a Natural Population
Source: PLoS One. 2008 Mar 5;3(3):e1739. doi: 10.1371/journal.pone.0001739 (PMC2254494; doi:10.1371/journal.pone.0001739)
Supplement: Table S2 — Sample sizes for trait correlations and genetic covariances among seven morphological traits in the great reed warbler. (0.05 MB DOC) [file pone.0001739.s002.doc]

Table S2. Sample sizes (a) for trait correlations and genetic covariances (b) among seven morphological traits in the great reed warbler.

|  |  | *Wing length* | *Wing projection* | *Tail length* | *Bill width* | *Bill length* | *Skull length* | *Tarsus length* |
| --- | --- | --- | --- | --- | --- | --- | --- | --- |
| (a) Sample sizes for trait correlations^1)^ | | | | | | | | |
| Wing length |  |  | 375 | 376 | 376 | 379 | 308 | 456 |
| Wing projection |  | 84 |  | 371 | 369 | 372 | 302 | 371 |
| Tail length |  | 83 | 82 |  | 371 | 373 | 302 | 371 |
| Bill width |  | 82 | 80 | 80 |  | 374 | 304 | 372 |
| Bill length |  | 85 | 83 | 82 | 81 |  | 308 | 374 |
| Skull length |  | 64 | 62 | 61 | 61 | 64 |  | 303 |
| Tarsus length |  | 115 | 82 | 81 | 81 | 83 | 62 |  |
| (b) Genetic covariances^2)^ | | | | | | | | |
| Wing projection |  | 0.77 |  |  |  |  |  |  |
| Tail length |  | 1.98 | 0.42 |  |  |  |  |  |
| Bill width |  | 0.01 | 0.01 | 0.02 |  |  |  |  |
| Bill length |  | 0.003 | 0.04 | 0.11 | 0.02 |  |  |  |
| Skull length |  | 0.06 | 0.06 | 0.26 | 0.01 | -0.01 |  |  |
| Tarsus length |  | 0.002 | 0.13 | 0.001 | 0.02 | 0.09 | 0.08 |  |

1. Sample sizes corresponds to the number of parent-offspring comparisons used for calculating the genetic correlation (under the diagonal) and the number of individuals in the pedigree measured for both traits (above the diagonal).
2. The genetic covariances (b) are calculated from repeated measures animal model.
